# Red blood cell distribution width is associated with sarcopenia risk in early-stage non-small cell lung cancer

**DOI:** 10.1186/s12885-024-11864-z

**Published:** 2024-01-17

**Authors:** Qing-chun Jia, Ling Qin, Ye Niu, Le Liu, Ping-ping Liu, Shi-di Miao, Ming-ming Cui, Rui-tao Wang

**Affiliations:** 1 Department of Internal Medicine, Harbin Medical University Cancer Hospital, Harbin Medical University, NO.150 Haping ST, Nangang District, Harbin, Heilongjiang 150081 China; 2 Department of Pathology, Harbin Medical University Cancer Hospital, Harbin Medical University, Harbin, Heilongjiang 150081 China; 3 School of Computer Science and Technology, Harbin University of Science and Technology, Harbin, Heilongjiang 150080 China

**Keywords:** Red blood cell distribution, Sarcopenia, Non-small cell lung cancer, Computed tomography

## Abstract

**Background:**

Sarcopenia has received increasing attention in non-small cell lung cancer (NSCLC). Red blood cell distribution width (RDW) is a significant component of the complete blood count and indicates the heterogeneity of erythrocyte volume. Little information is known about RDW in relation to sarcopenia in early-stage (IA-IIIA) NSCLC. The purpose of the present study was to investigate the association between RDW and sarcopenia risk in early-stage NSCLC patients.

**Methods:**

This study included 378 patients with pathologically confirmed stage IA-IIIA NSCLC. Sarcopenia was defined by measuring the skeletal muscle index (SMI) at the eleventh thoracic vertebra level. The maximum Youden index on the receiver operating characteristic (ROC) curve was used to estimate the cutoff value for RDW to predict sarcopenia. Logistic regression analyses were carried out to assess the independent risk factors for sarcopenia in NSCLC.

**Results:**

The ROC curve indicated that the best cutoff point for RDW to predict sarcopenia was 12.9 (sensitivity of 43.80% and specificity of 76.76%, respectively). Moreover, there were significant differences in hemoglobin (*p* < 0.001), comorbidities (*p* = 0.001), histological type (*p* = 0.002), and cancer stage (*p* = 0.032) between the high RDW and low RDW groups. Logistic regression analyses revealed that high RDW is an independent risk factor for sarcopenia in early-stage NSCLC.

**Conclusion:**

RDW is associated with sarcopenia risk in early-stage NSCLC.

## Introduction

Globally, lung cancer (LC) is one of the deadliest cancers [1]. Non-small cell lung cancer (NSCLC) is the most prevalent pathological type of LC, accounting for more than 80% of patients [2]. Researchers have found that the prognosis of LC is not only related to cancer characteristics, such as cancer stage, but also to other factors, such as sarcopenia [3]. Sarcopenia is defined as a syndrome characterized by a significant loss of muscle strength [4]. The incidence of sarcopenia in LC patients ranges from 46 to 79% [5]. Previous studies have found that in NSCLC, esophageal, bladder, and ovarian cancer, patients diagnosed with sarcopenia before surgery have a poorer prognosis than those without sarcopenia [6].

The skeletal muscle area (SMA) on CT images at the third lumbar vertebra level (L3) has been used as one of the standard methods for the diagnosis of sarcopenia because of its good correlation with systemic skeletal muscle mass [7]. However, in clinical work, chest CT does not include the image at the L3 level. Some studies have found that the SMA of the eleventh thoracic vertebra level (T11) can also be used as an indicator to reflect the skeletal muscle of the whole body [8].

Red blood cell distribution width (RDW) is one of the red blood cell (RBC) parameters, and its significance is the variation of circulating erythrocyte size [9]. Many studies have demonstrated that RDW is an independent factor in the poor prognosis of LC [10, 11]. Koma Y. et al. confirmed that RDW was correlated with the clinical stage of cancer, and the higher the RDW value, the worse the prognosis for LC patients [12]. In addition, RDW can help distinguish benign from malignant colon tumors [13].

Few studies have examined the relationship between RDW and the risk of sarcopenia in early-stage (IA-IIIA) NSCLC. Accordingly, this study aims to investigate whether RDW is associated with sarcopenia in patients with early-stage NSCLC.

## Methods

### Participants

**Fig. 1:**
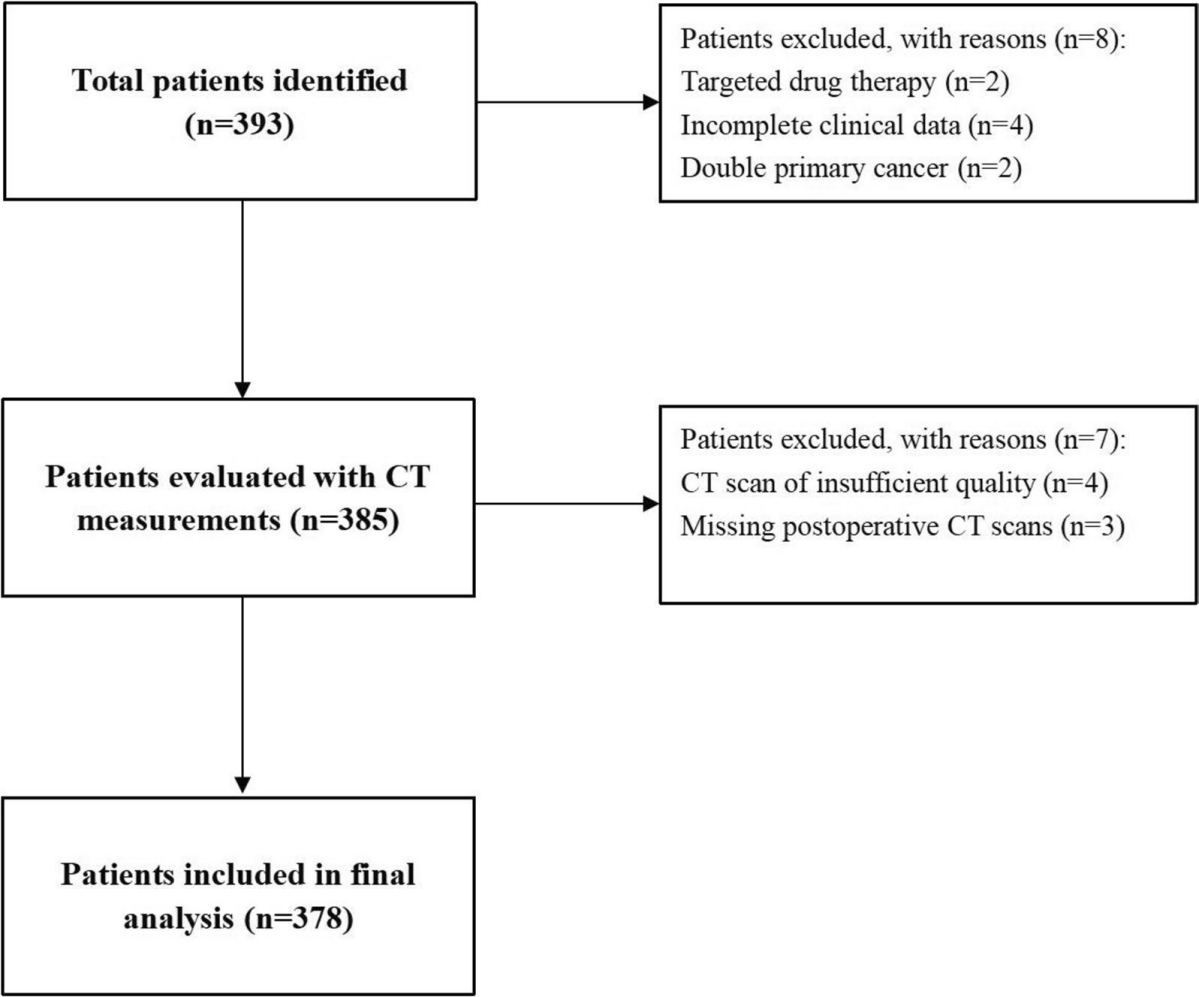
Flow chart for patient inclusion

We collected data about the patients who underwent lobectomy for early-stage NSCLC in the database of the Harbin Medical University Cancer Hospital from 2020 to 2021. The eligibility criteria were as follows: (i) patients over the age of 18 with pathologically confirmed NSCLC; (ii) without distant metastasis at diagnosis; (iii) patients who have not received anti-tumor therapy. Exclusion criteria: (i) patients merged with other types of tumors or hematological and autoimmune diseases; (ii) respiratory failure; (iii) insufficient clinical data or pre-operative CT images were not available. 378 patients were enrolled in this study (Fig. 1).

This study protocol was approved by the Ethics Committee of the Harbin Medical University Cancer Hospital. Since it was a retrospective study, informed consent from all participants was exempted.

### Laboratory measurements

All patients fasted for 8 h before blood collection to measure their complete blood count, including white blood cell (WBC), platelet count (PLT), RDW, and hemoglobin. The normal RDW reference range in our laboratory is 11.5-16.5%. In addition, the neutrophil to lymphocyte ratio (NLR), platelet to lymphocyte ratio (PLR), and prognostic nutritional index (PNI) were calculated. PNI is calculated as serum albumin (ALB) (g/L) + 5×total lymphocyte count (10^9^/L) [14].

### Calculation of sarcopenia

The SMA was obtained from the CT images at T11 level by Image J software (National Institute of Health, Bethesda, MD, USA). Tissue segmentation was based on pre-established thresholds of HU in the range of − 29 to + 150 [15]. All skeletal muscle area measurements were performed by two technicians using a double-blind method, and SMA was defined as the mean of two measurements. Skeletal muscle index (SMI) was calculated by dividing the SMA by the height squared (cm^2^/m^2^). Because SMI varies by sex and race, the threshold for diagnosing sarcopenia with T11-level SMI on CT images has not been clearly defined. Sex-specific cut-off values at the lowest tertile for SMI were calculated to diagnose sarcopenia [16–18].

### Statistical analysis

The categorical variables are expressed as numbers and percentages. Mean ± standard deviation (SD) is used to report normally distributed continuous data. For continuous variables, the two groups were divided to compare the significant difference using the Student’s t-test or the Mann-Whitney U test. For the categorical variables, the Chi-square test was used. The cutoff value for RDW to predict sarcopenia was estimated using the maximum Youden index value on the receiver operating characteristic (ROC) curve. Logistic regression analysis was performed to determine the risk factors for sarcopenia. The Pearson correlation coefficient was applied to the correlation analyses between SMI and RDW. MedCalc version 15.0, and SPSS Statistics version 26.0 were used for statistical analysis. A *p*-value < 0.05 represents statistical significance.

## Results

**Fig. 2:**
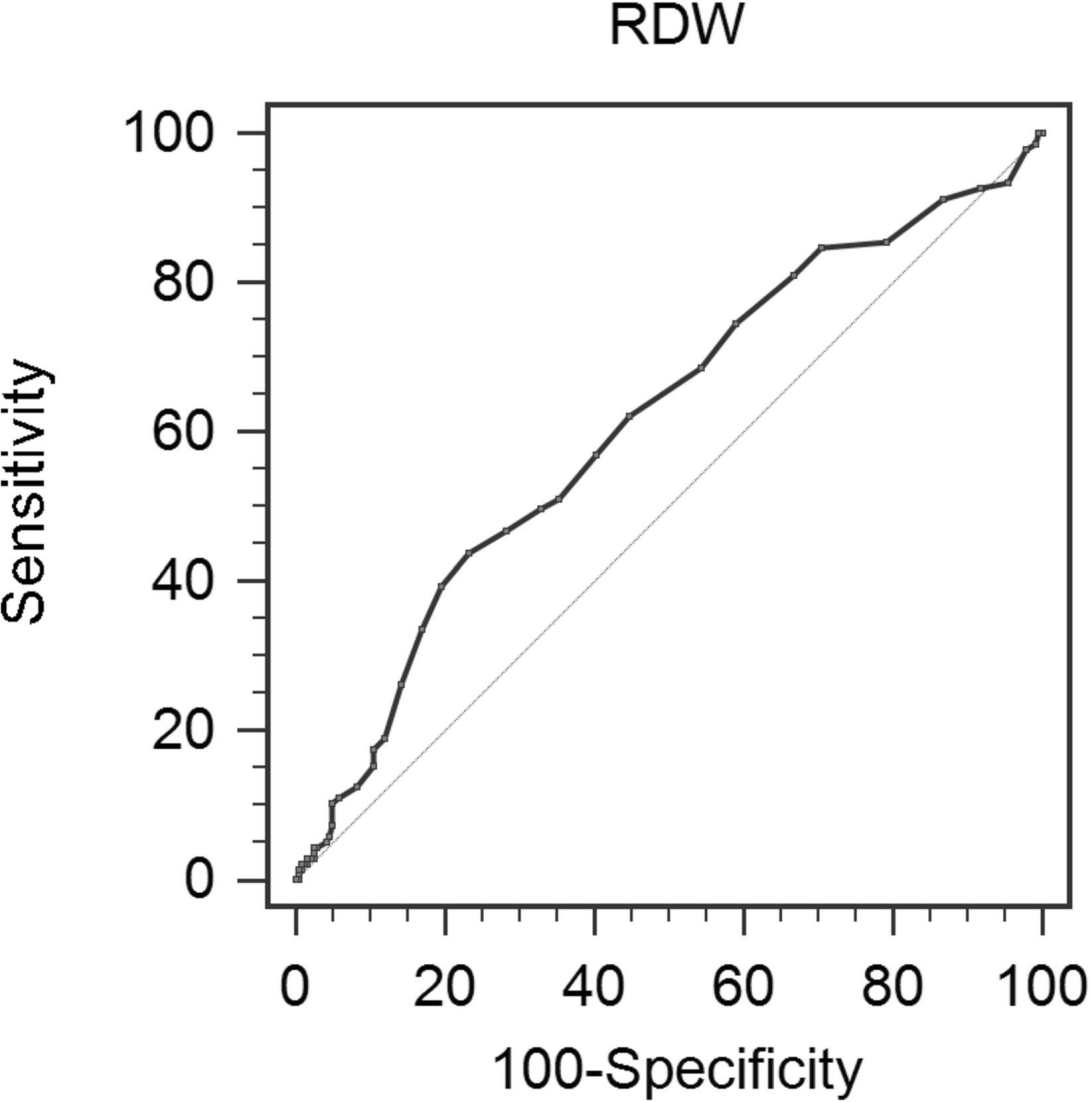
An optimized cut-off value was determined for RDW using ROC curve analysis

According to the ROC, RDW > 12.9 is the optimal cutoff point to discriminate the risk of sarcopenia, with an AUC of 0.613 (95% confidence interval (CI) = 0.562–0.662, *p* = 0.0002), a sensitivity of 43.80%, and a specificity of 76.76% (Fig. 2).

**Fig. 3:**
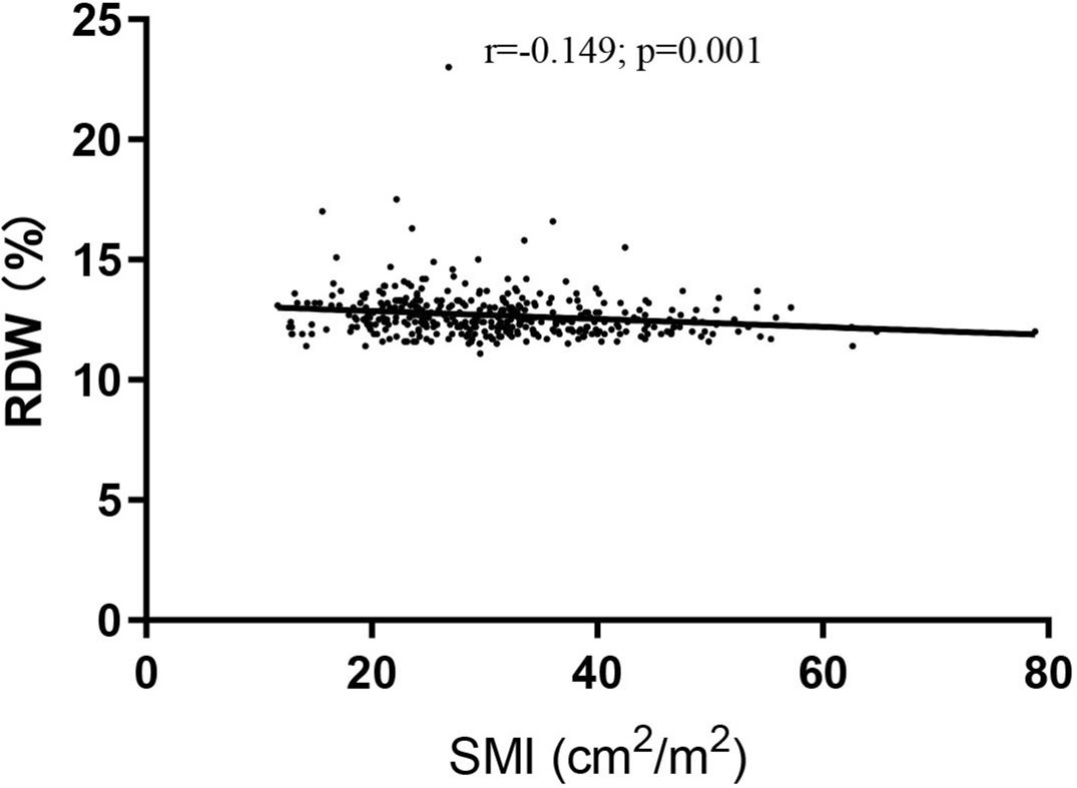
The partial correlation between RDW and SMI

The baseline characteristics of NSCLC patients according to RDW status are presented in Tables 1 and 2. We observed no differences between the groups in age, gender, BMI, current smoker, pack-years, current drinker, hypertension, diabetes, tumor size, T stage, lymph node status, WBC, platelet count, NLR, PLR, PNI, and albumin. However, there were significant differences in histological type (*p* = 0.002), comorbidities (*p* = 0.001), cancer stage (*p* = 0.032), and hemoglobin (*p* < 0.001) between the two groups. Additionally, those patients with high RDW have lower SMA and SMI levels than those with low RDW. The prevalence of sarcopenia in the high-RDW group was higher than that in the low-RDW group (51.7% vs. 29.4%, *p* < 0.001). A significant correlation was detected between RDW and SMI (*p* = 0.001, *r* = -0.171) (Fig. 3).

**Table 1:** Baseline characteristics of NSCLC patients according to RDW status

Variables	Total	RDW ≤ 12.9	RDW > 12.9	*p*-value
	n (%)	n (%)	n (%)	
Age (years)				0.240
≤ 60	219 (57.9)	157 (59.9)	62 (53.4)	
> 60	159 (42.1)	105 (40.1)	54 (46.6)	
Gender				0.604
Male	177 (46.8)	125 (47.7)	52 (44.8)	
Female	201 (53.2)	137 (52.3)	64 (55.2)	
Current smoker				0.441
Yes	104 (27.5)	69 (26.3)	35 (30.2)	
No	274 (72.5)	193 (73.7)	81 (69.8)	
Pack-years of smoking	8.9 ± 17.4	9.0 ± 16.8	8.9 ± 17.7	0.941
Current drinker				0.727
Yes	275 (72.8)	70 (26.7)	33 (28.4)	
No	119 (44.1)	192 (73.3)	83 (71.6)	
Hypertension				0.054
Yes	75 (19.9)	59 (22.5)	16 (13.9)	
No	302 (80.1)	203 (77.5)	99 (86.1)	
Diabetes				0.135
Yes	47 (12.4)	37 (14.1)	10 (8.6)	
No	331 (87.6)	225 (85.9)	106 (91.4)	
Comorbidities				0.001
Yes	184 (48.7)	142 (54.2)	74 (63.8)	
No	194 (51.3)	120 (45.8)	42 (36.2)	
Tumor size				0.969
> 4cm	55 (14.6)	38 (14.5)	17 (14.7)	
≤ 4cm	323 (85.4)	224 (85.5)	99 (85.3)	
Histological type				0.002
AD	319 (84.4)	88 (75.9)	231 (88.2)	
SCC	59 (15.6)	28 (24.1)	31 (11.8)	
T stage				0.960
T1+T2	345 (91.3)	239 (91.2)	106 (91.4)	
T3+T4	33 (8.7)	23 (8.8)	10 (8.6)	
Lymph node status				0.592
Negative	255 (67.5)	179 (68.3)	76 (65.5)	
Positive	123 (32.5)	83 (31.7)	40 (34.5)	
Cancer stage				0.032
I/II	305 (80.7	219 (83.6)	86 (74.1)	
III	73 (19.3)	43 (16.4)	30 (25.9)	
Sarcopenia				< 0.001
Yes	137 (36.2)	77 (29.4)	60 (51.7)	
No	241 (63.8)	185 (70.6)	56 (48.3)	

**Table 2:** Baseline characteristics of NSCLC patients according to RDW

Variables	Total	RDW ≤ 12.9	RDW > 12.9	*p*-value
Age (years)	58.0 ± 8.4	57.9 ± 8.4	58.3 ± 8.3	0.631
BMI (kg/m^2^)	24.1 ± 3.3	24.3 ± 3.2	23.7 ± 3.4	0.091
WBC (×10^9^/L)	6.54 ± 2.11	6.42 ± 1.87	6.80 ± 2.56	0.160
Hemoglobin (g/dl)	142.8 ± 14.3	144.8 ± 13.2	138.4 ± 15.8	< 0.001
Platelet count (×10^9^/L)	240.8 ± 69.4	238.0 ± 68.2	247.2 ± 72.1	0.234
NLR	2.26 ± 2.58	2.03 ± 1.16	2.78 ± 4.29	0.066
PLR	132.4 ± 73.9	127.8 ± 49.6	142.9 ± 110.2	0.160
Albumin (g/L)	41.9 ± 3.2	42.0 ± 3.1	41.5 ± 3.5	0.192
PNI	52.0 ± 4.9	52.1 ± 4.7	51.6 ± 5.3	0.334
SMA (cm^2^)	84.5 ± 29.9	88.4 ± 30.3	75.9 ± 27.3	< 0.001
SMI (cm^2^/m^2^)	31.0 ± 10.3	32.4 ± 10.5	27.8 ± 9.0	< 0.001

*RDW* Red blood cell distribution width, *AD* Adenocarcinoma, *SCC* Squamous cell carcinoma, *BMI* Body mass index, *NLR* Neutrophil-to-lymphocyte ratio, *PLR* Platelet-to-lymphocyte ratio, *RDW* Red blood cell distribution width, *PNI* Prognostic nutritional index, *SMA* Skeletal muscle area, *SMI* Skeletal muscle index

The correlation coefficient between RDW and SMI was − 0.171 (*p* = 0.001) (Fig. 3). After adjusting for age, sex, BMI, smoking status, alcohol consumption, hypertension, diabetes, tumor size, histological type, T stage, lymph node status, cancer stage, WBC, hemoglobin, platelet count, and albumin, the partial correlation coefficient between RDW and SMI was − 0.149 (*p* = 0.005).

The risk factors found to be associated with sarcopenia in the univariate logistic regression analysis were age, BMI, and RDW. These parameters were included in the multivariate logistic regression analysis model. The results identified that age (years) (odd ratio (OR) 1.033, 95% CI: 1.005–1.062, *p* = 0.019), BMI (OR 0.900, 95% CI: 0.839–0.966, *p* = 0.003), and RDW (OR 1.324, 95% CI: 1.053–1.664, *p* = 0.016) were the statistically significant risk factors. In particular, RDW is an independent factor for sarcopenia (Table 3).

**Table 3:** The risk factors for sarcopenia based on logistic regression

Variables	COR (95% CI)	*p*-value	AOR (95% CI)	*p*-value
Age (>60 vs ≤60 years)	1.624 (1.063 – 2.483)	0.025	1.646 (1.059 – 2.557)	0.027
Gender (male vs female)	0.865 (0.568 – 1.318)	0.499		
BMI (≥24.0 vs <24.0 kg/m^2^)	0.571 (0.373 – 0.873)	0.010	0.551 (0.355 – 0.855)	0.008
Smoker (yes vs no)	1.078 (0.675 – 1.720)	0.754		
Drinker (yes vs no)	1.039 (0.650 – 1.663)	0.872		
Hypertension (yes vs no)	0.848 (0.497 – 1.447)	0.546		
Diabetes (yes vs no)	0.896 (0.471 – 1.705)	0.737		
Comorbidities (yes vs no)	0.670 (0.439 – 1.023)	0.063		
Histological type (AD vs SCC)	1.055 (0.593 – 1.875)	0.856		
Lymph node status (positive vs negative)	0.873 (0.556 – 1.371)	0.556		
Cancer stage (III vs I+II)	1.119 (0.661 – 1.895)	0.676		
Hemoglobin (≥110 vs <110 g/dl)	0.419 (0.092 – 1.901)	0.260		
WBC (≥7.0 vs <7.0 ×10^9^/L)	1.452 (0.935 – 2.255)	0.097		
Platelet count (≥200 vs <200 ×10^9^/L)	1.316 (0.811 – 2.137)	0.266		
RDW (≥12.9 vs <12.9 %)	2.574 (1.640 – 4.041)	< 0.001	2.516 (1.591-3.981)	< 0.001
Albumin (≥35 vs < 35 g/L)	0.368 (0.102 – 1.329)	0.127		

The crude odds ratio (COR) with 95% confidence interval (CI) in univariate analysis and adjusted odds ratio (AOR) with (95% CI) in multivariate analysis were shown for variables with significance

*BMI* Body mass index, *AD* Adenocarcinoma, *SCC* Squamous cell carcinoma, *NLR* Neutrophil-to-lymphocyte ratio, *PLR* Platelet-to-lymphocyte ratio, *RDW* Red blood cell distribution width, PNI Prognostic nutritional index

## Discussion

The main observation of the study was that RDW is a risk factor for sarcopenia in early-stage NSCLC. Higher RDW values were related to a higher sarcopenia risk.

The molecular mechanism by which RDW is involved in sarcopenia remains unclear. However, inflammation plays a key role in the development of sarcopenia. Previous studies have demonstrated that inflammatory responses occur during the incidence and progression of cancer [19]. Sarcopenia molecular pathways are hypothesized to be activated by a sustained overexpression of proinflammatory mediators [20]. Sarcopenia is associated with interleukin-6 (IL-6) and C-reactive protein (CRP), which are markers of inflammation [21]. CRP can also predict preoperative muscle loss [15]. In one study, it was discovered that RDW, CRP, and erythrocyte sedimentation rate are all significant indicators of inflammation. RDW has a positive correlation with CRP, tumor necrosis factor-α, and IL-6, but a negative correlation with IL-10 [22]. By reducing muscle anabolism and supply-demand balance, IL-6 has been demonstrated to cause muscular atrophy. It may also be involved in modulating muscle catabolism [23]. Increased IL-6 inhibits the maturation of RBC in the bone marrow, causing immature RBC to enter the circulation and resulting in elevated RDW [24]. A report observed that muscle strengthening activities were associated with RDW, according to a cohort analysis of 8257 US participants [25]. In patients with solid cancers, patients at either early or advanced stages had an inverse relationship between high pretreatment RDW and poor OS [26]. The correlation between increased RDW and reduced overall survival has been confirmed at any stage of NSCLC [12]. Therefore, inflammation may contribute to the link between RDW and sarcopenia in NSCLC.

Some studies have reported that age, BMI, and platelet count are risk factors for sarcopenia [27, 28]. Another study found a correlation between increased RDW and sarcopenia in general American adults [20]. These are consistent with our study. However, there was no statistical significance for some inflammatory indicators in our study, such as NLR, WBC, and PLR. This is different from the results of some previous reports. Borges TC et al. found a correlation between NLR and sarcopenia risk in cancer patients [29]. WBC was found to be independently associated with sarcopenia in a Korean study [30]. Lin J et al. demonstrated that preoperative NLR and PLR are the independent predictors of sarcopenia in gastric cancer [31]. The reasons are speculated to be related to differences in race, sample size, and criteria for inclusion and exclusion of patients.

The study has certain limitations. First, we only included patients with early-stage NSCLC, which affects the general applicability of the results. Second, the inflammatory marker, such as CRP, was not analyzed in this retrospective study. We will consider incorporating CRP into future studies to enhance the comprehensiveness of our research. Third, the possibility of over-fitting may exist due to the relatively small sample size in this study. Finally, continued investigation of the mechanism of action is warranted.

Early detection of sarcopenia in cancer is a challenge in clinical practice. RDW is a simple, cost-effective, and noninvasive diagnostic biomarker. Our study demonstrated the prognostic value of RDW in resectable NSCLC. Future prospective studies are required to confirm our findings and expand the prognostic utility of RDW to other types of cancer. Moreover, it would be interesting to investigate whether adding RDW to sarcopenia prognosis scores could improve their performance.

In conclusion, RDW is associated with sarcopenia risk in early-stage NSCLC. RDW may help to detect sarcopenia early in NSCLC patients. Exploring its underlying molecular mechanism will be helpful in seeking potential therapeutic targets.

## Data Availability

The data are available from the corresponding author upon request.

## Abbreviations

LC: Lung cancer
NSCLC: Non-small cell lung cancer
SMA: Skeletal muscle area
L3: The third lumbar vertebra level
T11: The eleventh thoracic vertebra level
RDW: Red blood cell distribution width
RBC: Red blood cell
WBC: White blood cell
PLT: Platelet count
NLR: Neutrophil to lymphocyte ratio
PLR: Platelet to lymphocyte ratio
PNI: Prognostic nutritional index
SMI: Skeletal muscle index
SD: Standard deviation
ROC: Receiver operating characteristic
CI: Confidence interval
OR: Odd ratio
IL-6: Interleukin-6
CRP: C-reactive protein
